# Breast biopsies and breast cancer risk in Israeli *BRCA* germline pathogenic variant carriers

**DOI:** 10.1007/s10549-025-07787-3

**Published:** 2025-07-22

**Authors:** Dana Madorsky Feldman, Miri Sklair-Levy, Yael Laitman, Renata Faermann, Noam Nissan, Osnat Halshtok Neiman, David Samoocha, Yael Yagil, Eitan Friedman

**Affiliations:** 1 Meirav High-Risk Clinic, Sheba Medical Center, Tel-Hashomer, Ramat Gan, Israel; 2 Division of Diagnostic Imaging, Sheba Medical Center, Tel-Hashomer, Ramat Gan, Israel; 3 The Oncogenetics Service, Assuta Medical Center, Ramat Hachayal, Tel Aviv, Israel; 4 Tel-Aviv University School of Medicine, Tel Aviv, Israel

**Keywords:** BRCA PV carriers, Breast biopsy, Proliferative and non-proliferative benign breast disease, Breast cancer, Atypia, Histological instability

## Abstract

**Purpose:**

Benign breast disease (BBD), particularly with proliferative changes, is a risk factor for breast cancer (BC) development in average risk women. There is a paucity of data on high-risk, *BRCA1* and *BRCA2* pathogenic variants (PVs) carriers.

**Methods:**

Female *BRCA1* and *BRCA2* PV carriers treated at the Meirav Clinic, Sheba Medical Center between May 2011 and December 2024 were eligible. Data on in-hospital breast biopsies were retrieved following an ethically approved protocol. Statistical analyses included *χ*^2^ test (categorical variables) Mann–Whitney *U* test (continuous variables) and logistic regression for multivariate analysis.

**Results:**

Overall, 1466 women (849 *BRCA1* PV carriers) were monitored over 10,113 women/years. A total of 1453 biopsies were carried out in 454 participants (range 1–8 biopsies), with the majority (76.3%) benign and 242 (16.6%) malignant. Rates of BC in women undergoing at least two benign biopsies were correlated with the number of biopsies, being an older *BRCA1* PV carrier, whereas having been diagnosed with fibroadenoma—seems not to increase BC risk.

**Conclusions:**

In Israeli *BRCA* PV carriers, the number of biopsies, *BRCA1* PV carriership were associated with an increased risk for developing BC, whereas fibroadenoma does not increase that risk. It is imperative to validate these preliminary observations.

## Introduction

Breast cancer (BC) is the most common malignancy among women globally, with more than 2 million women affected annually in 2022 [1] and 5384 cases reported in Israel in 2020 (https://www.gov.il/en/pages/23102022-01). The lifetime risk of developing BC in the general average risk population is ~ 13.1%, with several risk factors associated with an increased risk, including age over 50 years, a family history of cancer, reproductive and hormonal influences, and inherited predisposition syndromes (https://www.cancer.org/content/dam/cancer-org/research/cancer-facts-and-statistics/breast-cancer-facts-and-figures/2024/breast-cancer-facts-and-figures-2024.pdf). Women harboring germline pathogenic variants (PV) in the *BRCA1* (MIM# 113705) and *BRCA2* (MIM# 600185) genes have a substantially elevated lifetime risk of BC, ranging from 69 to 72% [2]. Benign breast diseases (BBD), encompass a range of non-malignant breast lesions, varying from non-proliferative to proliferative lesions, with or without atypia. BBD involving proliferative alterations and atypical hyperplasia has been reported to increase the risk of BC compared to BBD without proliferative histological features. Hartmann et al. [3] quantified the relative risk of BC at 1.88 for proliferative disease without atypia and 4.24 for atypical hyperplasia based on an analysis of 9087 women with BBD over a median surveillance period of 15 years. Ashbeck et al. [4] reported that women with BBD had an almost twofold increase in risk—hazard ratio (HR 1.95) for developing BC compared with those without BBD. Dyrstad et al. [5] reviewed 32 publications analyzing BBD associated BC risk and concluded that proliferative disease without atypia was associated with a significantly increased risk of BC, relative risk (RR) of 1.76, and for atypical hyperplasia, RR was 3.93. Lohani et al. [6] showed that elevated RR of BC (1.87) were similar for Hispanic (*n* = 3684) and non-Hispanic women (*n* = 6587) diagnosed with BBD, with the risk of BC more pronounced with increasing severity of the BBD. These observations imply that the presence of specific BBD conditions, rather than the biopsy procedure per se, could be indicative of an elevated subsequent risk of BC. Notably, there is a paucity of data to assess the impact on the BC risk of BBD in women classified as high-risk based on family history, PV carrier status in a cancer susceptibility gene (mainly *BRCA1* and *BRCA2*), or polygenic risk score (PRS). Sherman et al. [7] reported that women with BBD in the highest PRS tertile exhibit a greater than twofold increase in BC odds ratio (OR = 2.73) relative to the reference PRS group with the lowest tertile. Zeinomar et al. [8] studied 1208 *BRCA1/BRCA2* PV carriers and concluded that the BC risks associated with BBD were not affected by *BRCA* mutational status.

To further elucidate the possible impact of BBD on BC risk among *BRCA* PV carriers, we assessed the impact of the number of breast biopsies and the diversity of histological types of BBD on subsequent BC risk within a cohort of Israeli *BRCA1* or *BRCA2* PV carriers, followed in a high-risk clinic in a single medical center in Israel.

## Methods

All women enrolled in the Meirav High-Risk Clinic, located at Sheba Medical Center, Tel-Hashomer, Israel, from May 2011 to December 2024, were included in this study. All participants were confirmed carriers of *BRCA1* and/or *BRCA2* germline PVs prior to their clinic enrolment. Genotyping was carried out following formal oncogenetic counseling for several indications: women diagnosed with BC cancer, unaffected family members as cascade testing after identifying *BRCA* PV in a first or second degree family member, cancer-free individuals who are members of high-risk families predicated to have 20% or greater likelihood for developing BC by established algorithms, such as BRCAPRO (https://projects.iq.harvard.edu/bayesmendel/brcapro), Tyrer Cusick (https://magview.com/ibis-risk-calculator/), and BOADICEA (later named CanRisk—https://www.canrisk.org/), or in accordance with clinically applied guidelines (e.g., https://www.nccn.org/guidelines/category_2). As of 2020, genotyping for the predominant Jewish Ashkenazi *BRCA* PVs became embedded in the Israeli MoH Health Basket as population screening tool without the need for pre-test counseling (https://call.gov.il/product-page/10010967) and subset of participants was the result of this population screen. All Israeli female carriers of *BRCA* PVs are offered a surveillance scheme for the early detection of BC from ages 25 to 80 years: biannual clinical breast examinations by a surgeon combined with biannual breast imaging options alternating MRI with ultrasound (for ages 25 to 30 years) or mammography (for ages 31 to 80 years). The data documented in the Chamelon Intra-hospital Electronic Health Records (EHR) system and retrieved securely and de-identified utilizing MDclone (https://mdclone.com/) included: date of birth, date at genotyping, clinic joining date, the mutated *BRCA* gene, previous and current cancer diagnoses and dates, laterality, number and histological reports of all in-hospital biopsies conducted. This study was performed in line with the principles of the Declaration of Helsinki. Approval was granted by the Ethics Committee of the SMC IRB (SMC 1492-14), and due to its nature, was exempted from acquiring individualized specific informed consent for each participant, who had previously provided umbrella consent for such de-identified data analyses at the time of clinical enrollment. Histopathological grades of benign lesions were categorized into five distinct groups as follows. Normal breast tissue—labelled 0; non-proliferative lesions [fibrosis, simple and lactating cysts, adenosis (non-sclerosing), duct ectasia, hamartoma, neurofibroma, granuloma, lipoma] were labelled 1; advanced non-proliferative lesions without atypia (ductal hyperplasia, benign phyllodes, pseudo angiomatous stromal hyperplasia, fibroadenoma)—labeled 2; proliferative lesions without atypia (sclerosing adenosis, florid ductal hyperplasia with apocrine metaplasia, multiple papillomas)—labeled 3; proliferative lesions with atypia (mainly Atypical ductal or lobular hyperplasia—ADH, ALH)—labelled 4; malignant histology was labelled 5 (for ductal carcinoma in situ—DCIS and lobular carcinoma in situ—LCIS) and 6 (for invasive ductal carcinoma IDC).

### Statistical analysis

All statistical analyses were conducted using R (version 4.3.1) and SPSS (version 29.0). A structured analytical pipeline was implemented to assess associations between the number of breast biopsies, progression of benign histology, and the risk of breast cancer diagnosis in *BRCA1/2* PV carriers. Descriptive statistics were used to summarize demographic and clinical characteristics. Temporal progression from non-proliferative to proliferative benign lesions across serial biopsies was assessed using repeated-measures logistic regression. Group differences were analyzed using the *χ*^2^ test for categorical variables and the Mann–Whitney *U* test for non-normally distributed continuous variables. Time-to-event analyses, were performed using Kaplan–Meier survival curves and compared using the log-rank test. Spearman’s rank correlation coefficient (Spearman *ρ*) was calculated to assess monotonic relationships between the number of breast biopsies and the likelihood of subsequent cancer diagnosis. Multivariate logistic regression models were employed to estimate odds ratios (ORs) and 95% confidence intervals (CIs) for breast cancer diagnosis, controlling for covariates. All statistical tests were two-sided, and a *p*-value < 0.05 was considered statistically significant. Multivariate logistic regression analysis was carried out using standardized continuous variables (age at joining, number of biopsies, follow-up years), and gene mutation type (*BRCA1* vs. *BRCA2*) as predictors for BC development.

## Results

### Participant characteristics

A total of 1466 women were eligible—849 (57.9%) *BRCA1* PV carriers, 607 (41.4%) *BRCA2* PVs carriers, and 10 (0.6%) with PVs in both genes. The median age at genotyping was 34.3 years for *BRCA1* PV carriers and 37.1 years for *BRCA2* PV carriers (*p* = 0.0001), and mean age at joining the clinic was 41.8 ± 12.7 years, median 39.3 years for all women. The median duration of follow-up was 8.25 years, with 987 women (67.8%) being followed-up for at least 5 years (10,113 women-years). The most frequent *BRCA1* PV was c.68_69del [p.Glu23fs, also known as (aka) 185delAG], identified in 606 (71.4%) *BRCA1* PV carriers, while 142 (16.7%) carried the c.5266dup (p.Gln1756fs, aka 5382insC) variant. The prevalent *BRCA2* PV was c.5946del (p.Ser1982fs, aka 6174delT), observed in 543 (88.6%) *BRCA2* PV carriers. Combined, these three “founder Jewish” PVs [9] accounted for 88.7% of all participants.

### Risk reducing surgery

Risk reducing mastectomy (RRM) was performed by 169 (11.5%) women total: 128 *BRCA1* carriers (mean age 38.2 ± 7.25 years; median 37.8 years) and 41 *BRCA2* carriers (38.4 ± 6.77 years; median 37.4 years, *p* = 0.84 for age difference). Risk reducing salpingo-oophorectomy (RRSO) was performed by 922 (65.6%) women: 558 *BRCA1* carriers (mean age at surgery 55 ± 11.24 years; median 52.2 years) and 364 *BRCA2* carriers (mean age 58.5 ± 11.05 years; median 56.7 years, *p* = 2.3 × 10^−6^). Five women who have had RRM (all *BRCA1* carriers) developed BC after RRM: the interval from RRM to the post-mastectomy cancer ranged 2.5 to 12.7 years (mean ≈ 6.5 years, median ≈ 3.0 years).

### BC diagnoses

Overall, 181 *BRCA1* PV carriers were diagnosed with BC prior to clinic entry at a mean age of 42.3 ± 11.0 years, median 40.9 years, and of *BRCA2* PV carriers, 87 were diagnosed with BC before clinic entry at a mean age of 48.7 ± 11 years (*p* = 0.00015). Of these women—33/268 (12.3%, 25 *BRCA1* carriers) developed second BC, the mean time to second BC was 11.84 ± 7.85 years (range 1.5–31.7 years). No significant difference between *BRCA1* and *BRCA2* PV carriers in age at second diagnosis (*p* = 0.93).

### Biopsy details

Overall, 454/1456 (31.2%) participants (10 double PV carriers excluded, 280 *BRCA1* carriers) had a total of 1453 biopsies (841 in *BRCA1* carriers). Of biopsied *BRCA1* carriers each woman had a mean 3.22 ± 1.41 biopsies (range 1–8), and for *BRCA2* carriers—mean 2.94 ± 1.37 biopsies (range 2–6, *p* = 0.094).

**Fig. 1:**
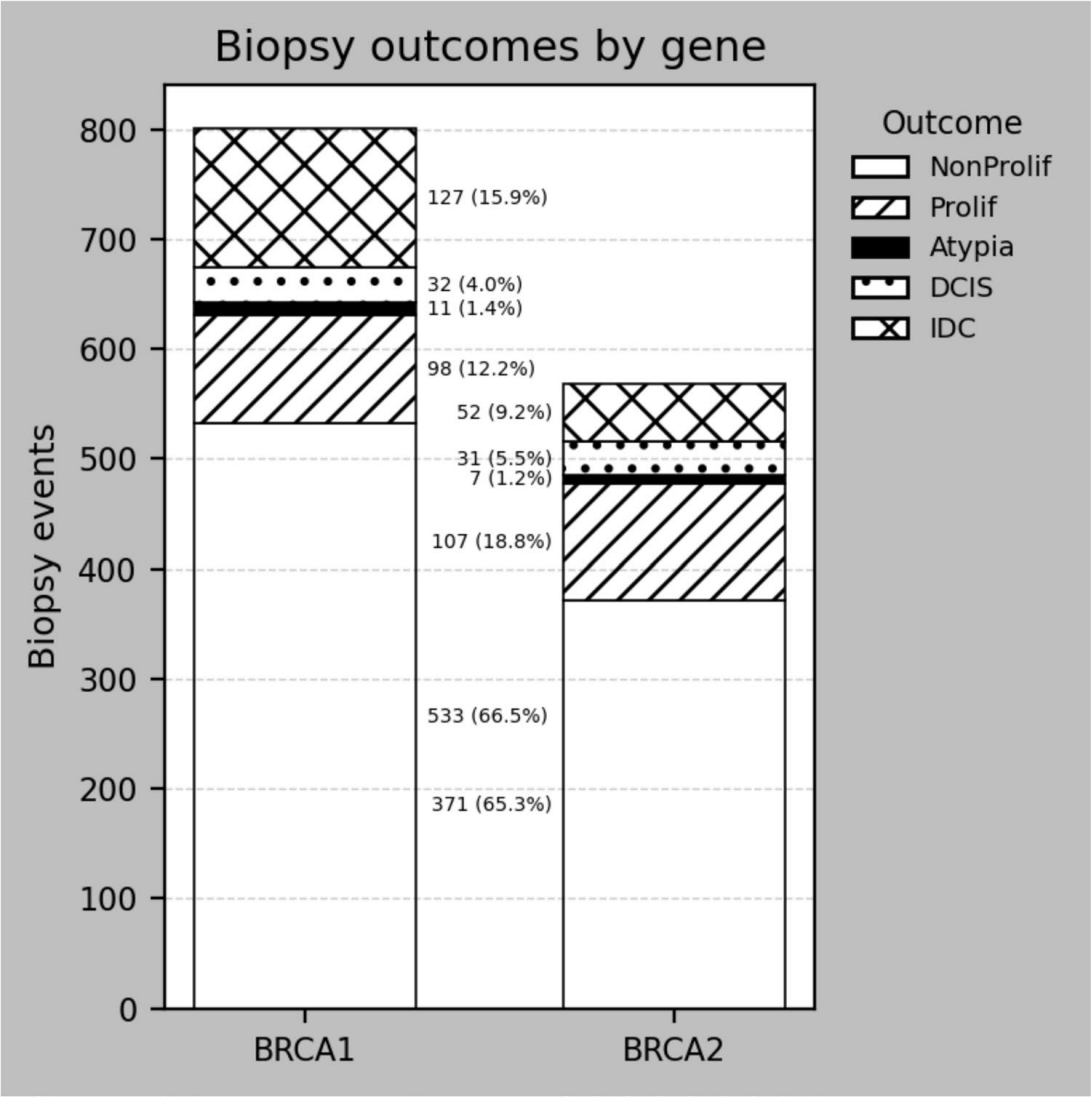
Benign and malignant lesion distributions by biopsy and by mutated gene. Biopsy histological outcomes by gene. The numbers and % indicate the number of the total individuals and % of the total of individuals with the relevant histological subtypes diagnosed in the entire study

Of biopsies, 1109 (631 *BRCA1*, 76.3%) were benign, 242 (16.6%, 159 *BRCA1*) were malignant, 18 (11 *BRCA1*, 1.6%) had atypia (along with other benign proliferative changes) and 102 (7%) did not contain breast tissue (muscle, skin, lymph node, fat). Proliferative benign changes were more common in *BRCA2* carriers (11.7% *BRCA1* vs. 17.5% *BRCA2*; *p* = 0.004) whereas IDC was significantly more common in *BRCA1* (15.2% vs. 8.5%, *p* = 0.0004). Figure 1 displays the range of benign histological biopsy outcomes by gene.

Of women who did not undergo RRM and had at least two benign biopsies (*n* = 251; 134 *BRCA1* carriers) a change in histological patterns—from normal breast tissue (grade 0), non-proliferative histology (grades 1 and 2) and proliferative changes (grades 3 and 4) was analyzed to see if any changes—progression or regression—were associated with subsequent BC risk. Of these, 70% (*n* = 175) remained non-proliferative throughout biopsies, 5% (*n* = 12) remained proliferative with no regression to non-proliferative. Additionally, 34 (13.5%) progressed from non-proliferative to proliferative and 30 (11.9%) regressed from proliferative to non-proliferative. BC was diagnosed in this cohort in 70 women (50 *BRCA1* carriers). There was no evidence that stability or change (progression or regression) affects later BC risk when both genes are pooled (*χ*^2^ = 0.58, *p* = 0.45). Gene specific analysis did not alter that conclusion (*p* ~ 0.63 and ~ 0.72 for *BRCA1* and *BRCA2* respectively). Additionally, there was no association between the side of the breast that had more biopsies and the side of the breast where eventually BC was diagnosed in 149 women (*χ*^2^ = 0.005, *p* ≈ 0.94) with no change per gene.

**Fig. 2:**
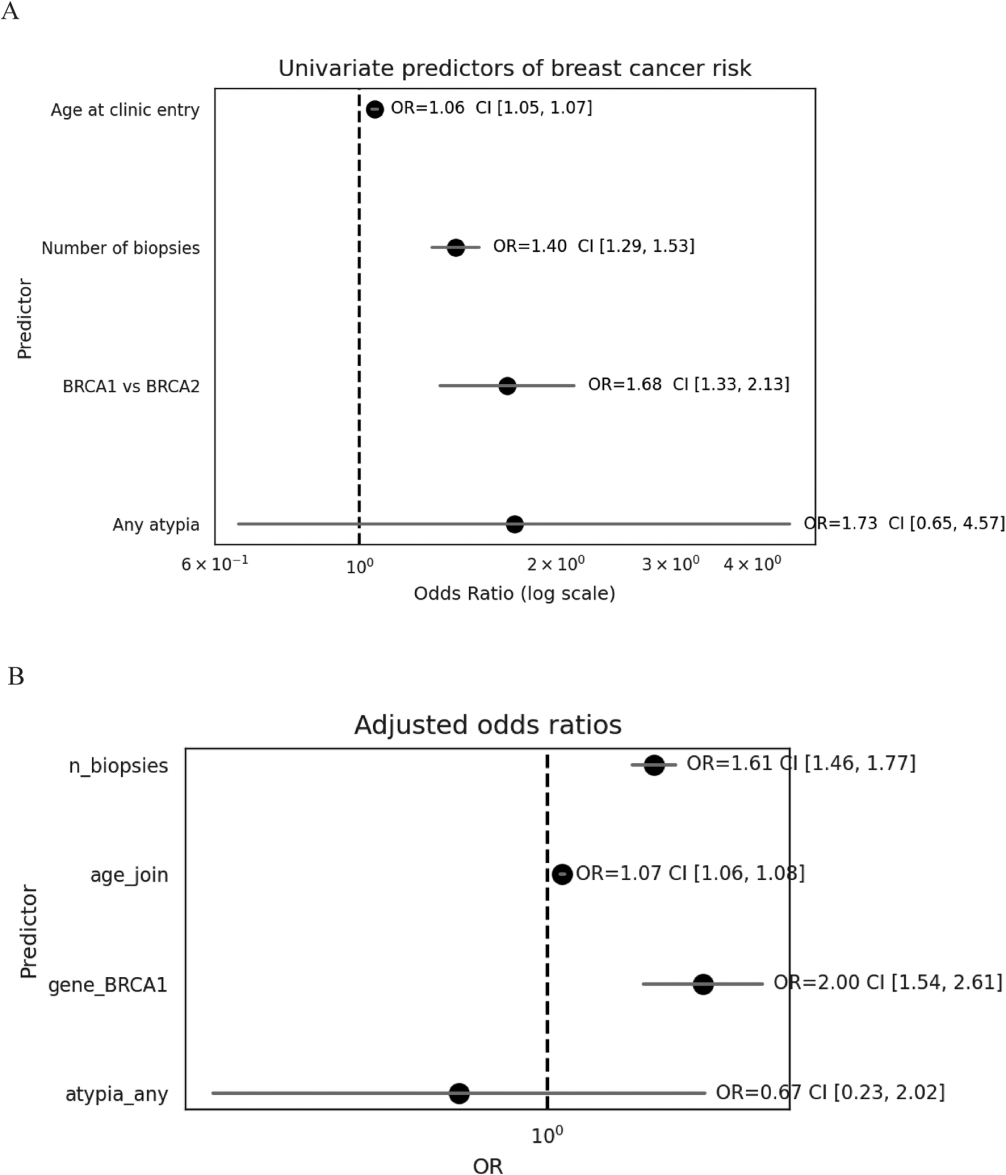
**A** Univariate analysis of the factors associated with prediction of BC risk in women who have had at least two benign biopsies. **B** Multivariate analysis of the same cohort

Univariate analysis to define parameters predominantly associated with predicting BC risk in *BRCA* carriers was applied to women who did not undergo RRM and have had at least two benign breast biopsies (mean biopsy number 2.76; *n* = 251; 134 *BRCA1* PV carriers) of whom 70 (27.9%) eventually developed BC. In this cohort the best predictors of BC development were having multiple biopsies (OR = 1.40, CI [1.29, 1.53]; *p* < 10^−14^), age at joining the clinic (OR = 1.06 per year, CI [1.05, 1.07]; *p* < 10^−28^) and being *BRCA1* carrier (*BRCA1* vs. *BRCA2*, OR = 1.68, CI [1.33, 2.13]; *p* = 0.001). The presence of atypia in any biopsy was associated with elevated yet statistically insignificant higher rates of BC (OR = 1.73, CI [0.65, 4.57]). Similar results with the same statistical significance were obtained applying multivariate analysis, and this model performed reasonably (AUC ≈ 0.76, Fig. 2).

Fibroadenoma (FA) was diagnosed in 235 participants (134 *BRCA1* carriers and 101 *BRCA2* carriers). BC was diagnosed in 26.9% of *BRCA1* FA cases and in 12.9% of *BRCA2* FA cases. These rates were significantly lower compared with rates in *BRCA2* carriers who did not have FA diagnosed with BC (24.9%, *p* ≈ 0.013), and for all *BRCA* carriers combined—FA diagnosis has shown an independent protective effect (OR ≈ 0.21, CI [0.10–0.44], *p* < 0.001).

**Fig. 3:**
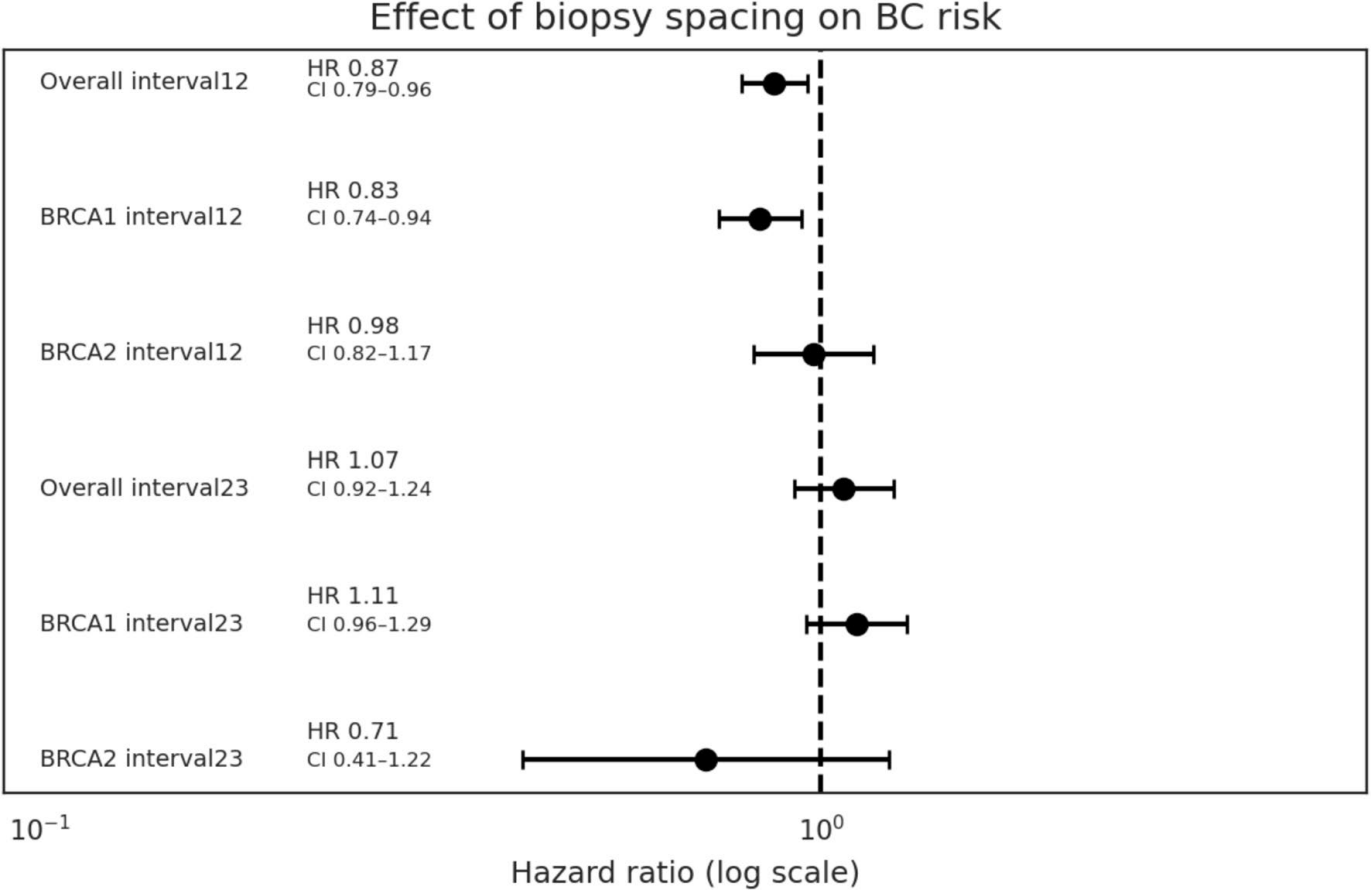
Time elapsed between first and second biopsy and *interval12—time (years) between the first and second biopsy; *interval23—time (years) between the second and third biopsy

Analysis of the effect of the time that elapsed (in years) between the first and second benign biopsy and the second and third benign biopsies showed that for every year between the two first biopsies, BC risk is lowered by 13% (HR 0.87; *p* ~ 0.005). No such risk lowering effect was noted for the time between the second and third biopsies (HR 1.07, *p* ≈ 0.37). The major contributor to this BC lowering effect is being a *BRCA1* PV carrier [Interval12 remains significant (HR 0.83, *p* ≈ 0.004), Fig. 3].

## Discussion

In the present study, the total number of benign breast biopsies and being an older BRCA1 PV carrier increased BC risk in a large cohort of Israeli BRCA PV carriers followed up for a combined of more than 10,000 women-years. Uniquely in the current study, having proliferative BBD and atypia were not associated with BC risk, no consistent pattern of benign histological progression from non-proliferative to proliferative changes associated with BC development and fibroadenoma was not associated with an increased risk for developing BC. Compared with previously reported studies focused on high-risk cohorts and female *BRCA* PV carriers, there are some consistent and some inconsistent observations. Increased BBD-associated BC risk correlated with proliferative changes with or without atypia in high-risk women [10], but not in those diagnosed with fibroadenoma [11]. Yet, Nassar et al. [12] reported increased BC risk in women with multiple first-degree relatives diagnosed with fibroadenoma. Collins et al. [13] reported that BBD exhibiting proliferative lesions without atypia and a positive family history were at a significantly elevated risk of developing BC compared with those with non-proliferative lesions and no family history (OR = 2.45). Visscher et al. [14] reported that among 1414/13,466 women with biopsy-confirmed BBD, those exhibiting progression from non-proliferative to proliferative findings were at a higher risk of developing BC (HR 1.77; *p* = 0.03) compared to those who displayed no progression. Sherman et al. [7] analyzed data from five Breast Cancer Association Consortium (BCAC, https://www.ccge.medschl.cam.ac.uk/breast-cancer-association-consortium-bcac) case–control studies involving 6706 BC cases and 8488 controls and reported that women with BBD and the highest tertile BC polygenic risk score (PRS) had more than twice the odds for developing BC (OR = 2.73), compared to the reference group. Burke et al. [15] studied the factors associated with BC risk among women diagnosed with BBD, and reported that a positive family history—typically having one first-degree relative with BC was correlated with BC risk in all BBD subsets.

Notably, these previous studies did not stratify BC risk by *BRCA* mutational status, but rather assigned BC risk mostly phenotypically by the number of affected family members and age at diagnosis or by PRS. Zeinomar et al. [8] in a study comprising 1208 *BRCA1/BRCA2* PV carriers, reported that the risks associated with having BC attributed to BBD were unaffected by *BRCA* mutational status, with an increased risk for *BRCA1* carriers (HR 1.64) and *BRCA2* carriers (HR 1.34), similar to non-carriers (HR 1.31, *p*_interaction_ = 0.95). Several factors may account for the different findings of the current study compared with some of those of previous reports. Firstly, cancer risks in *BRCA* PV carriers are partially related to the specific location of the PV along the gene [16]. As most *BRCA* PV carriers in the present study harbored one of the three predominant *BRCA* PVs common in Jewish individuals, all located within the respective breast cancer cluster region (BCCR) of these two genes, the risk for BC and potentially even benign precursors might differ from those reported by Zeinomar et al. [8]. Another possibility to account for some of the divergent results in the current study from previous ones may stem from the specific indications for biopsy (mass on clinical breast exam or a suspicion raised by an abnormal breast imaging finding). The lack of association between atypia and BC risk in the current study may actually reflect the rarity of these findings rather than a true biological phenomenon. Interestingly, Fibroadenoma in any benign biopsy is associated with a 47% lower hazard of breast cancer (HR ≈ 0.53, *p* ≈ 1 × 10^−4^), and that reduced risk remained significant after adjusting for age, biopsy burden, and gene. These results contrast the results reported by Nassar et al. [12], though no *BRCA* genotyping was reported in that study. The observed time interval between the first and second biopsies as a determinant of BC risk may actually reflect the biology of tumor development where a slower growth rate (as reflected by the biopsy indication) combined with the heightened surveillance scheme. It is premature to try and incorporate this parameter in a multivariate scheme that can potentially be used to better stratify BC risk in *BRCA* PV carriers.

Uni and multivariate analyses show that total number of biopsies, age, and being a BRCA1 gene PV carrier exhibit the strongest positive association with BC development, likely indicating both increased surveillance in high-risk individuals and the higher rates of BC risks in *BRCA1* vs. *BRCA2* PV carriers. Interestingly, maximum benign histological grade and proliferative changes did not show increased BC risk in the current study, possibly indicating that more subtle, tissue specific factors underlie BC development that are not reflected by a simple distinction between “proliferative” and “non proliferative” changes.

This study has certain limitations. It is a single-center study and may not represent all *BRCA* PV carriers in Israel and certainly not in ethnically diverse, genetically heterogeneous populations, as it predominantly involves individuals harboring one of the three *BRCA* PVs. Furthermore, additional BC risk factors (e.g., oral contraceptive use, parity, breastfeeding, physical activity, body mass index) or additional genetic modifiers [17] that may alter BC risk in *BRCA* PV carriers were not stratified in this study. Additionally, the primary rationale for performing a biopsy, which may have confounded the results, was not taken into consideration.

In conclusion, among Israeli *BRCA* PV carriers, a correlation between the number of breast biopsies being an older *BRCA1* PV carrier and subsequent BC risk was noted, with fibroadenoma not being a forerunner of BC risk in *BRCA* carriers. It also raises the possibility that the mere distinction between “proliferative” and “non proliferative” BBD changes maybe an insufficient tool to predict future BC risk in *BRCA* PV carriers It is essential for these preliminary findings to be further validated in larger, ethnically diverse future studies.

## Data Availability

The datasets generated during and/or analyzed during the current study are not publicly available due to participant confidentiality reasons but are available as an anonymized aggregate format from the corresponding author on reasonable request.
